# Plasma SHBG Levels as an Early Predictor of Response to Bariatric Surgery

**DOI:** 10.1007/s11695-023-06981-w

**Published:** 2024-01-06

**Authors:** P. Gabriel-Medina, R. Ferrer-Costa, F. Rodriguez-Frias, M. Comas, R. Vilallonga, A. Ciudin, D. M. Selva

**Affiliations:** 1 Clinical Biochemistry Department, Vall d’Hebron University Hospital, 08035 Barcelona, Spain; 2 Biochemistry and Molecular Biology Department, Universitat Autònoma de Barcelona (UAB), 08193 Barcelona, Spain; 3 Biochemical Chemistry, Drug Delivery & Therapy (BC-DDT) Research Group, Vall d’Hebron Institut de Recerca (VHIR), 08035 Barcelona, Spain; 4 Centro de Investigación Biomédica en Red de Enfermedades Hepáticas y Digestivas (CIBEREHD), 28029 Madrid, Spain; 5 Endocrinology and Nutrition Department, Vall d’Hebron University Hospital, Pg Vall d’Hebron 119-129, 08035 Barcelona, Spain; 6 Endocrine, Metabolic and Bariatric Unit, Center of Excellence for the EAC-BC, Vall d’Hebron University Hospital, Universitat Autònoma de Barcelona, Barcelona, Spain; 7 Diabetes and Metabolism Research Unit, Diabetes and Metabolism Department, Vall d’Hebron Institut de Recerca (VHIR), Universitat Autònoma de Barcelona (UAB), Pg Vall d’Hebron 119-129, 08035 Barcelona, Spain; 8 Centro de Investigación Biomédica en Red de Diabetes y Enfermedades Metabólicas Asociadas (CIBERDEM), 28029 Madrid, Spain

**Keywords:** Bariatric surgery, Sex hormone-binding globulin, Biomarker, Weight loss, Weight regain

## Abstract

**Background:**

Obesity is a growing global health problem, and currently, bariatric surgery (BS) is the best solution in terms of sustained total weight loss (TWL). However, a significant number of patients present weight regain (WR) in time. There is a lack of biomarkers predicting the response to BS and WR during the follow-up. Plasma SHBG levels, which are low in obesity, increase 1 month after BS but there is no data of plasma SHBG levels at long term. We performed the present study aimed at exploring the SHBG role in predicting TWL and WR after BS.

**Methods:**

Prospective study including 62 patients with obesity undergoing BS. Anthropometric and biochemical variables, including SHBG were analyzed at baseline, 1, 6, 12, and 24 months; TWL ≥ 25% was considered as good BS response.

**Results:**

Weight loss nadir was achieved at 12 months post-BS where maximum SHBG increase was reached. Greater than or equal to 25% TWL patients presented significantly higher SHBG increases at the first and sixth months of follow-up with respect to baseline (100% and 150% respectively, *p* = 0.025), than < 25% TWL patients (40% and 50% respectively, *p* = 0.03). Also, these presented 6.6% WR after 24 months. The first month SHBG increase predicted BS response at 24 months (OR = 2.71; 95%CI = [1.11–6.60]; *p* = 0.028) and TWL in the 12th month (*r* = 0.330, *p* = 0.012) and the WR in the 24th (*r* =  − 0.301, *p* = 0.028).

**Conclusions:**

Our results showed for the first time that increase in plasma SHBG levels within the first month after BS is a good predictor of TWL and WR response after 2 years.

**Graphical Abstract:**

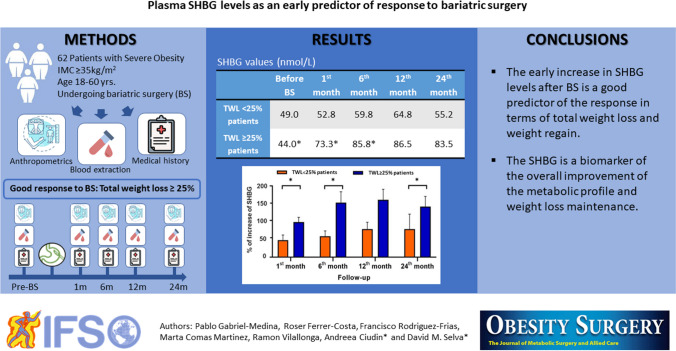

**Supplementary Information:**

The online version contains supplementary material available at 10.1007/s11695-023-06981-w.

## Introduction

Obesity is a global health epidemic leading to the development of metabolic syndrome (MetS), type 2 diabetes mellitus (T2D), metabolic-associated fatty liver disease (MAFLD), dyslipemia, and cardiovascular disease (CVD) [1, 2] among other serious complications. Obesity and its associated comorbidities have been exponentially increased during the last 40 years [3]. In this regard, the adult population worldwide with obesity and overweight is 13% and 39% respectively [4], exceeding the 24% of prevalence for obesity in Europe and the USA [5]. Obesity management represents a medical and socio-economic burden in industrialized and in developing countries [6].

Obesity leads to adipose tissue inflammation which contributes to the development of peripheral and hepatic insulin resistance (IR) and MAFLD [7]. Furthermore, obesity gives rise to increased intestinal permeability, resulting in higher circulating levels of microbiome antigens, which amplify inflammatory processes and proinflammatory adipokines dysregulation [8]. This dysregulation potentially leads to metabolic disorders and chronic complications such as CVD, hypertension, and systemic IR [9]. Another effect of this adipokine imbalance is the downregulation of the biosynthesis of liver proteins, such as sex hormone-binding globulin (SHBG). In this regard, it has been described that pro-inflammatory cytokines decrease, and anti-inflammatory cytokines increase hepatic SHBG production, respectively [10–12]. The role of SHBG as a biomarker associated with metabolic dysregulation has been described in experimental animal models, which have shown that glucose- and fructose-induced lipogeneses decrease liver SHBG synthesis [13]. In addition, plasma SHBG levels have been inversely correlated with intrahepatic fat content, IR, and body mass index (BMI) [14, 15], so it is considered a biomarker for MetS [16] and predictive of T2D [17] and CVD [18, 19].

At present, bariatric surgery (BS) represents the best solution in terms of sustained weight reduction and remission of the associated metabolic comorbidities in patients with obesity [20]. European guidelines recommend BS to be considered for patients of 18–60 years with BMI ≥ 40.0 kg/m^2^ or BMI ≥ 35.0 and comorbidities expected to improve after significant weight loss [21–23]. Long-term response to BS can be variable and was usually evaluated by % excess weight loss (EWL) and % total weight loss (TWL). Classically, EWL > 50% and more recently TWL ≥ 25% cut-offs have been defined as “good response” to BS [24]. Nevertheless, weight regain (WR) occurs in a significant number of patients after BS [25]. Previous studies have reported that the nadir EWL and TWL after BS were maintained in < 50% and < 25%, respectively, after 20 years of follow-up [26]. A recent meta-analysis reported that 17.6% of patients who underwent BS had a WR ≥ 10% starting 3 years after BS [27]. The WR etiology is multifactorial. Several factors have been proposed to explain WR, including pre-operative BMI, hormonal factors, nutrition habits, physical activity, mental health, genetics [28, 29], and anatomical changes [30]. Nevertheless, these factors do not completely explain the WR after BS [31].

To the best of our knowledge, there is no reliable biomarker that predicts successful TWL and/or WR after BS. Overall, BS restores metabolic homeostasis [32]; apart from TWL and reduction in waist circumference (WC), there is an increase in SHBG levels on the first month after BS, accompanied by the secretion of anti-inflammatory and insulin-sensitizing factors [33–35]. On these bases, we designed the present study to explore the impact of BS on SHBG, as well as the potential role of SHBG as reliable biomarker for predicting TWL and WR after BS in patients with obesity.

## Method

### Study Design and Patients

A prospective study, including consecutive patients with obesity attended at the Obesity Unit of the Vall d’Hebron University Hospital (VHUH) that underwent BS from June 2018 to January 2020, was performed. The study was conducted according to the Declaration of Helsinki and was approved by the local Ethics Committee (PR(AG)320/2018). Serum samples from patients included in this study were provided by the VHUH Obesity Biobank (PT17/0015/0047), integrated in the Spanish National Biobanks Network, and they were processed following standard operating procedures with the appropriate approval of the Ethical and Scientific Committees. All participants had previously signed the informed consent.

Inclusion criteria were as follows: (a) age, 18–60 years; (b) BMI ≥ 40 kg/m^2^ or BMI ≥ 35 kg/m^2^ with comorbidities; (c) preoperatory protocol fulfilment for BS; and (d) Roux-en-Y-gastric bypass (RYGB) or sleeve gastrectomy as BS technique.

Exclusion criteria were as follows: (a) BS contraindication according to the usual clinical practice in our center; (b) impossibility of performing the follow-up for at least 2 years; and (c) other BS techniques.

**Fig. 1:**
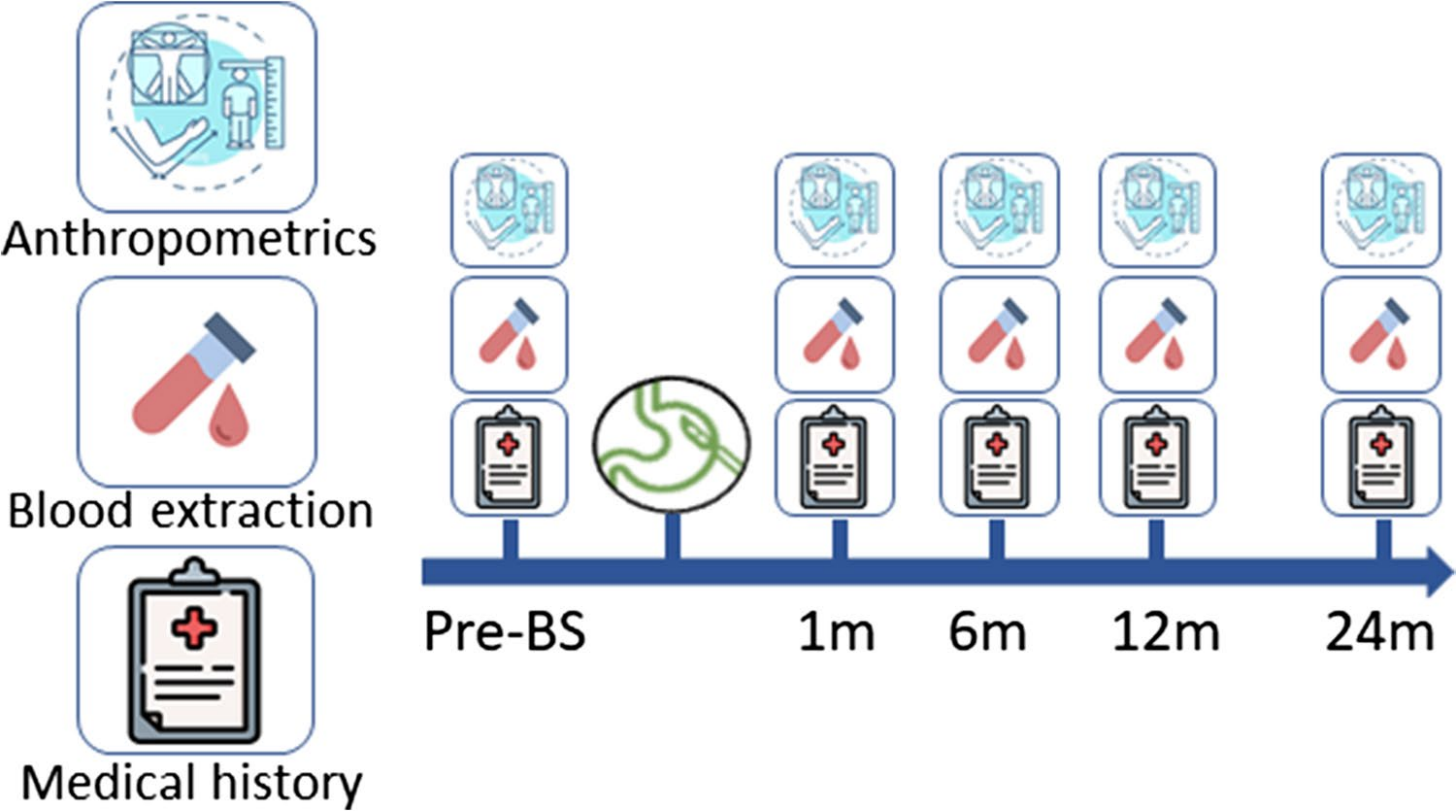
Study flowchart. Pre-BS, previous bariatric surgery follow-up; 1 m, 1st month after BS follow-up; 6 m, 6th month after BS follow-up; 12 m, 12th month after BS follow-up; 24 m, 24th month after BS follow-up

As per BS protocol at our site, patients are visited before BS (pre-BS), 1 month (1st month follow-up), 6 months (6th follow-up), 12 months (12th follow-up), and 24 months (24th follow-up) after BS. Anthropometry (weight, height, and waist circumference), biochemical analysis, and systematic extraction of blood samples in fasting conditions for Obesity Biobank are obtained at all-time points (Fig. 1). These data were used for the study.

### Clinical, anthropometric, and laboratory measurements

Weight loss was evaluated by TWL (%) calculation, as follows: 100*(weight (kg) at month follow-up/weight (kg) at pre-BS). A TWL cut-off ≥ 25% on the 24th follow-up was considered as good response to BS [24]. WR was calculated as [100*(post-nadir weight–nadir weight)]/nadir weight [36].

T2D was defined according to ADA guidelines [37]. Liver steatosis was measured by ultrasonography [38]. Hepatic IR was indirectly evaluated using the HOMA-IR, based on the formula: fasting glucose (mg/dl) × fasting insulin (μU/mL)/405 [39]. A cut-off ≥ 3.42 has been described as marker of IR in Caucasian population with obesity [40]. Patients with T2D on insulin treatment were excluded from the calculation of HOMA-IR.

SHBG levels (nmol/L) were measured using an enzyme-linked immunosorbent assay (ELISA) method (Demeditec Diagnostics GmbH®, Kiel, Germany) following manufacturer’s instructions (Intra assay CV = 2.3% and Inter assay CV = 5.2%).

### Statistical Analysis

The distribution of data was assessed by the Kolmogorov–Smirnov test. Student’s *T* test and Mann–Whitney *U* test were used to compare quantitative variables, which followed a Gaussian distribution or not, respectively. Paired samples *t* test or Wilcoxon test were used to compare each variable between follow-ups. A chi-squared test was used to compare proportions. Logistic regression analysis was performed to study the predictive ability of SHBG increase with response to BS. Multiple regression analysis was performed to assess the predictive ability of SHBG increase with the TWL on the 12th month and WR on the 24th month. Bonferroni test was used to discard outliers. All statistical analyses were performed with R-commander (R-UCA package v.2.6–2).

## Results

### Anthropometric and Biochemical Characteristics of the Study Cohort

A total of 62 patients fulfilling inclusion criteria with at least 2 years of follow-up were included; all of them were Caucasian. The 40% (*n* = 25) were diagnosed with T2D pre-BS. The treatment for T2D before BS were metformin (48%), insulin (16%), glucagon-like peptide-1 receptor agonists (GLP-1AR), sodium-glucose cotransporter-2 inhibitors (iSGLT2), or peroxisome proliferator-activated receptor gamma (PPARγ) agonists (12%); the rest (24%) were in diet alone.

T2D remission was observed in 76% of patients (52% females) with pre-BS T2D after 2 years of follow-up. At the first and sixth months, 52% of patients (36% females) with pre-BS T2D were suspended of drug therapy.

Furthermore, a 79% of patients showed liver steatosis, a 26% were under treatment for arterial hypertension and only the 8% were treated of hypothyroidism. None of the patients had a history of heart disease, anemia, liver cirrhosis, alcoholism, drug abuse, or mental disorders.

The main clinical and biochemical features of our cohort before BS are shown in Table 1. All patients followed the same pattern of lifestyle change, and the pharmacological treatments they were taking before surgery were not modified during the first month after BS; only two patients had contraceptive pill [41]. Weight, BMI, and waist circumference underwent a significant decrease reaching the nadir after 12 months in all patients (Table 2). The impact of BS on the biochemical parameters is shown in Table 2. Notably, the whole cohort presented an average pre-BS HOMA-IR greater than the cut-off considered normal for the Spanish population, and normalized from the first month after BS. Regarding SHBG, the blood levels increased significantly until the sixth month after BS, reaching the top at the 12th follow-up (Table 2).

**Table 1:** Clinical and biochemical variables pre-BS in the whole cohort and subdivided by gender

Variable	Pre-BS	Females	Males	*p* value
Age (years)	46 (10)	44 (10)	53 (8)	0.002
Sex, female	45 (73%)	-	-	-
Weight (kg)	120.6 (24.1)	114.2 (16.4)	137.6 (32.6)	< 0.001
BMI (kg/m^2^)	43.7 (6.9)	43.2 (6.0)	44.7 (9.5)	0.439
Waist circumference (cm)	126 (14)	120 (9)	137 (15)	< 0.001
Type 2 diabetes	25 (40%)	17 (38%)	8 (47%)	0.723
Fasting glucose (mg/dL)	105 (31)	98 (22)	123 (47)	0.009
HbA1c (%)	6.0 (1.1)	5.8 (0.9)	6.2 (1.3)	0.119
HOMA-IR	5.91 (4.20)	5.18 (3.82)	7.76 (4.57)	0.053
Triglycerides (mg/dL)	130 (99–167)	122 (89–157)	146 (122–171)	0.060
Total cholesterol (mg/dL)	198 (39)	198 (35)	198 (39)	0.945
Non-HDL cholesterol (mg/dL)	154 (35)	152 (35)	157 (35)	0.677
ALT (IU/L)	23 (16–36)	21 (14–36)	25 (20–32)	0.131
AST (IU/L)	21 (19–28)	20 (17–27)	22 (20–25)	0.263
GGT (IU/L)	31 (21–47)	27 (20–39)	45 (32–66)	0.007
SHBG (nmol/L)	51.0 (42.2)	48.5 (35.2)	37.9 (25.9)	0.267
Total testosterone (ng/dL)	-	29.3 (23.2–41.4)	293.1 (197.8–406.3)	< 0.001
Estradiol (pg/mL)	-	25.9 (17.6–56.7)	35.9 (26.4–41.2)	0.792
FIB-4	0.95 (0.57)	0.80 (0.40)	1.38 (0.73)	< 0.001
Platelets (× 10^9^/L)	292 (72)	302 (73)	264 (61)	0.076

Values are mean (standard deviation), number (%) or median (Q1–Q3)

*BS*, bariatric surgery; *BMI*, body mass index; *HbA1c*, glycated hemoglobin; *HOMA-IR*, homeostasis model assessment of insulin resistance; *HDL*, high-density lipoprotein; *ALT*, alanine aminotransferase; *AST*, aspartate aminotransferase; *GGT*, gamma-glutamyl transpeptidase; *SHBG*, sex hormone-binding globulin; *FIB-4*, fibrosis 4 index

**Table 2:** Clinical and biochemical variables along study

Variable	Pre-BS	1st month	6th month	12th month	24th month	*p* value pre-BS vs 1st	*p* value 1st vs 6th	*p* value 6th vs 12th	*p* value 12th vs 24th
Weight (kg)	120.6 (24.1)	107.8 (19.8)	89.2 (19.3)	82.4 (19.9)	83.0 (19.8)	< 0.001	< 0.001	< 0.001	0.402
BMI (kg/m^2^)	43.7 (6.9)	39.0 (5.9)	32.2 (6.1)	29.8 (6.1)	30.1 (6.3)	< 0.001	< 0.001	< 0.001	0.354
Waist circumference (cm)	126 (14)	117 (12)	101 (13)	96 (14)	96 (18)	< 0.001	< 0.001	< 0.001	0.536
Fasting glucose (mg/dL)	105 (31)	95 (21)	86 (12)	86 (13)	90 (18)	0.031	0.001	0.555	0.058
HbA1c (%)	6.0 (1.1)	5.7 (0.9)	5.1 (0.5)	5.2 (0.6)	5.4 (0.5)	< 0.001	< 0.001	0.185	0.001
HOMA-IR	5.91 (4.20)	3.17 (1.96)	1.82 (1.58)	1.69 (1.38)	1.92 (1.79)	< 0.001	< 0.001	0.251	0.092
Triglycerides (mg/dL)	130 (99–167)	138 (116–181)	94 (73–121)	83 (69–102)	79 (69–100)	0.051	< 0.001	< 0.001	0.312
Total cholesterol (mg/dL)	198 (39)	180 (32)	178 (35)	179 (32)	191 (39)	0.001	0.700	0.348	0.019
SHBG (nmol/L)	51.0 (42.2)	64.2 (47.8)	86.8 (72.4)	89.1 (64.6)	79.2 (52.8)	< 0.001	0.011	0.369	0.194

Values are mean (standard deviation), number (%) or median (Q1–Q3)

*BS*, bariatric surgery; *BMI*, body mass index; *HbA1c*, glycated hemoglobin; *HOMA-IR*, homeostasis model assessment of insulin resistance; *SHBG*, sex hormone-binding globulin

### BS Response and TWL Follow-up

Patients were subdivided according to TWL on the 12th month after BS, in < 25% TWL patients and ≥ 25% TWL patients, adjusted by age and gender-baseline data shown in Table 3. Both groups had the same proportion of females at menopause, and non-significant differences in glucose metabolism variables, SHBG levels, or any other parameters were observed before BS, included the surgical procedure (Table 3). Furthermore, the surgical procedure performed was independent of diabetes status (Supplementary Table 1).

**Table 3:** Clinical and biochemical basal characteristics in < 25% TWL patients versus ≥ 25% TWL patients

Variable	< 25% TWL patients (*n* = 20)	≥ 25% TWL patients (*n* = 42)	*p* value
Age (years)	45 (14)	47 8)	0.498
Sex, female	13 (65%)	32 (76%)	0.536
Menopause	4 (31%)	11 (34%)	0.907
Weight (kg)	121.6 (24.4)	120.1 (24.3)	0.818
BMI (kg/m^2^)	44.7 (8.0)	43.1 (6.6)	0.416
Waist circumference (cm)	127 (13)	124 (13)	0.346
T2D	11 (55%)	14 (33%)	0.167
Fasting glucose (mg/dL)	109 (44)	102 (24)	1.000
HbA1c (%)	6.0 (1.2)	5.8 (0.9)	0.533
HOMA-IR	6.45 (4.42)	5.47 (3.98)	0.427
SHBG (nmol/L)	49.0 (32.8)	44.0 (33.4)	0.581
RYGB technique	7 (35%)	27 (64%)	0.061

Values are mean (standard deviation) or number (%)

*BMI*, body mass index; *T2D*, type 2 diabetes; *HbA1c*, glycated hemoglobin; *HOMA-IR*, homeostasis model assessment of insulin resistance; *SHBG*, sex hormone-binding globulin; *RYGB*, Roux-en-Y-gastric bypass

The TWL at 6, 12, and 24 months after BS was greater in ≥ 25% TWL patients than in < 25% TWL patients (Table 4). At 24 months of follow-up, < 25% TWL patients underwent a significant average of 6.6% WR with respect to the nadir regardless of the surgical procedure.

**Table 4:** TWL (%) along the follow-up in < 25% TWL patients and ≥ 25% TWL patients

	1st month	6th month	12th month	24th month	*p* value 1st vs 6th	*p* value 6th vs 12th	*p* value 12th vs 24th
< 25% TWL patients	9.7 (8.4–11.0)	21.9 (19.3–24.6)	24.7 (21.8–27.7)	20.1 (17.7–22.4)	< 0.001	0.001	< 0.001
≥ 25% TWL patients	11.2 (10.0–12.3)	28.4 (26.5–30.2)	35.4 (33.8–37.0)	36.4 (34.4–38.3)	< 0.001	< 0.001	0.113
*p* value	0.116	< 0.001	< 0.001	< 0.001			

Values are mean (95%CI)

*TWL*, total weight loss

### Plasma SHBG Levels During the Follow-up in ≥ 25% TWL Patients and < 25% TWL Patients to BS

**Fig. 2:**
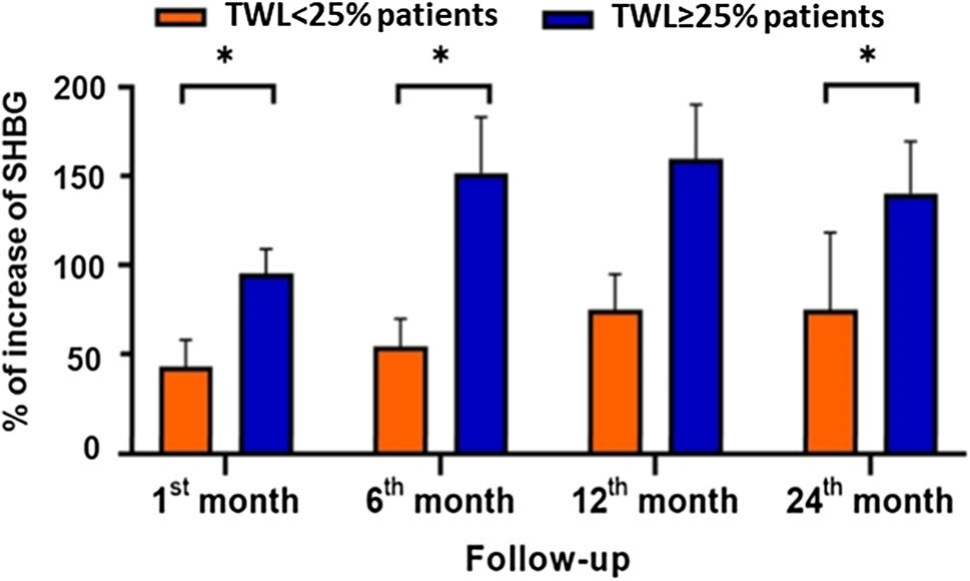
Mean percentages of SHBG increase and standard error (SE) in each follow-up with respect to SHBG value before BS in < 25% TWL patients and ≥ 25% TWL patients. *p < 0.050

Plasma SHBG levels were compared during the follow-up at different time points in both groups (Table 5). The plasma SHBG levels did not significantly increase in < 25% TWL patients 1 month after BS when compared with the initial levels (pre-BS); they increased around 13% at 6 months when compared with the SHBG levels at 1 month but there were no significant differences between 6 and 12 months or between 12 and 24 months. However, in ≥ 25% TWL patients, plasma SHBG levels increased significantly by 65% at 1 month when compared with pre-BS levels and by 16% between the first and sixth months, while no further increased was observed between the 6th and 12th months or the 12th and 24th months (Table 5). These results were corroborated by analyzing the SHBG variation (SHBG follow-up/SHBG pre-BS ratio) in both groups during the follow-up. The results showed that ≥ 25% TWL patients presented significantly higher increases of SHBG than < 25% TWL patients at each follow-up time point except on the 12th month (Fig. 2). On the other hand, no significant differences were observed in the increase of SHBG on the 1st month when considering the surgical procedure performed (Supplementary Table 2).

**Table 5:** SHBG values (nmol/L) along the follow-up in < 25% TWL patients and ≥ 25% TWL patients

	Before BS	1st month	6th month	12th month	24th month	*p* value pre-BS vs 1st	*p* value 1st vs 6th	*p* value 6th vs 12th	*p* value 12th vs 24th
< 25% TWL patients	49.0 (32.9–65.4)	52.8 (43.9–61.6)	59.8 (44.2–75.4)	64.8 (45.3–84.2)	55.2 (32.5–77.8)	0.621	0.090	0.272	0.581
≥ 25% TWL patients	44.0 (32.7–55.1)	73.3 (58.3–88.3)	85.8 (68.8–102.9)	86.5 (69.7–103.2)	83.5 (70.0–96.9)	< 0.001	0.022	0.698	0.197

Values are mean (95%CI)

### SHBG Plasma Levels as an Early Biomarker for TWL and WR

The logistic regression analysis showed an OR = 2.71 (95%CI = 1.11–6.60, *p* = 0.028) and AUC = 0.68 (95%CI = 0.55–0.80) for predicting good response to BS based on the SHBG 1st month/SHBG pre-BS ratio. Diagnostic performance parameters of different cut-off ratios assessed (expressed as percentages) are shown in Table 6.

**Table 6:** Parameters of diagnostic performance of SHBG increase on the 1st month with respect to pre-BS for predicting good response to BS

SHBG increase	Sensitivity (95%CI)	Specificity (95%CI)	PPV (95%CI)	NPV (95%CI)
48%	66.7% (49.8–80.9)	63.2% (38.4–83.7)	79.4% (61.7–91.4)	47.1% (26.7–68.3)
75%	56.4% (39.6–72.2)	79.0% (54.4–93.9)	85.1% (65.7–95.9)	46.0% (28.1–64.8)
82%	51.3% (34.8–67.6)	84.2% (60.4–96.6)	87.3% (66.9–97.4)	44.9% (27.8–62.8)

*SHBG*, sex hormone-binding globulin; *PPV*, positive predictive value; *NPV*, negative predictive value

Additionally, multiple regression analysis for predicting TWL on the 12th month after BS showed the following resultant model: TWL 12th month = 26.23 + 2.89 × SHBG 1st month/SHBG pre-BS (*r* = 0.330, *p* = 0.012). Furthermore, a prediction model for WR at 24 months was performed: resultant model WR 24th month = 3.30 − 1.72 × SHBG 1st month/SHBG pre-BS (*r* =  − 0.301, *p* = 0.028).

## Discussion

Patients with obesity have BS as the only solution in terms of a successful and sustained weight lost and improvement of the related metabolic comorbidities. Long-term response to BS can be variable, and WR occurs in a significant number of patients [20, 25], which means the reappearance or worsening of the associated comorbidities [27, 30, 42]. Therefore, there is a need for a biomarker to predict the TWL and WR in the long term after BS. Our results showed a 32% TWL at 12 months after BS. The TWL 1 year after BS observed in our study is similar to previous meta-analysis reporting an average TWL of 28–34% [36, 43]. Regarding the mean of WR in our study was 6.6% 1 year after nadir, and this percentage is comparable to the results of a previous study, where a 5.7% of WR was observed [36]. Although WR was calculated before the period recommended of 3 years, our mean did not differ much from the 8% reported previously [44]. In addition, the different trends in weight lost and regained observed in the 2 years of follow-up will be assessed in subsequent follow-ups.

Bariatric surgery improves metabolism in general and reduces the risk of obesity-associated disorders and all-cause mortality in patients with obesity [45–47]. In this sense, a T2D remission rate of 75% has been reported [48] similar to our results, in addition to a reduction of 70% in HOMA-IR along the first year [49]. Our results showed a HOMA-IR reduction from 5.91 to 1.69 in which the proposed HOMA-IR cut-off of 3.42 would inform us of an improvement in IR [40]. Furthermore, we also observed a significant reduction in fasting glucose and HbA1c levels along the first year after BS as described previously [45]. Regarding SHBG, our results showed that plasma SHBG levels increased after 1 month BS in all patients which has also been described previously in women with polycystic ovary syndrome and obesity [20] and recently reported in several meta-analysis, where SHBG rise from 25 to 130% [43, 50–52].

In order to assess if SHBG plasma levels were a reliable predictor for a good response to BS in terms of TWL and WR, we decided to measure plasma SHBG levels up to 24 months after BS. Our results showed for the first time that early plasma SHBG levels increased differently depending on the response to BS at 24 months. Remarkably, ≥ 25% TWL patients showed a mean increase of 100% in plasma SHBG levels on the 1st month, which yielded an increment of 150% 6 months after BS with respect to pre-BS values. However, < 25% TWL patients showed an increase of 40% in SHBG plasma levels on the 1st month, with SHBG relative increments at 6 to 24 months not higher than 75% with respect to pre-BS values. Importantly, the 1st month increase in plasma SHBG levels significantly predicted a TWL ≥ 25% to BS with a probability over 80%, regardless of age, gender, or surgical procedure. Furthermore, the 1st month SHBG increase also predicted the WR on the 24th month follow-up according to the multiple regression model. This increase in SHBG may be a consequence of decreased adipose tissue-related inflammation [53]. However, lifestyle modifications, such as fasting or exercise, have implications for the increasing SHBG expression, which could regulate energy expenditure [54–56]. Regarding surgical procedure, our data did not demonstrate that RYGB or the sleeve gastrectomy were associated with different outcomes in the response to BS along the follow-up, as previously reported [57], nor were they associated with a differential increase in SHBG on the first month after BS.

Thus, the early increase in SHBG observed in the ≥ 25% TWL patients could reflect the improvement of the metabolic profile in the medium and long term [58, 59], as previously reported, where WR is accompanied by an unfavorable metabolic profile [60]. Finding early post-BS biomarkers able to predict the mid- and long-term evolution is also interesting to identify, and thus complements the markers used prior to surgery, which are no-robust concerning BS response. This early identification of patients who will have WR 2 years after BS has a real potential of changing the current guidelines and would allow clinicians to conduct postoperative strategies and intensify treatments, such as behavior intervention, dietary counselling, and GLP-1AR in order to prevent WR [25, 61, 62]. The inclusion of SHBG in the analytical profiles of clinical practice and its accurate assessment would allow individualization in postoperative follow-up. Thus, collaboration between bariatric surgeons, obesity medicine specialists, and dietitians is required [63, 64], which would support the implementation of personalized medicine.

Our study has several limitations that should be noticed, such as (a) the absence of assessment of the relation between SHBG blood levels with basal metabolism and body composition change along the follow-up; (b) the influence of genetics in SHBG expression; (c) the role of adipokines variation after BS in the SHBG liver synthesis; and (d) the impact of each surgical technic used (RYGB and sleeve gastrectomy) in the BS response and WR, determined by the limited number of patients included. This is a pilot study, where future directions will be aimed at validating the cut-offs and predictive models obtained in a larger cohort considering all these variables and in determining a possible active role of SHBG in weight loss.

## Conclusion

The increase in plasmatic SHBG levels within the first month after BS is a good predictor of BS response in term of TWL and WR after 2 years of intervention. More studies are needed to elucidate the role of SHBG increase in the overall improvement of the metabolic profile and weight loss maintenance.

### Supplementary Information

Below is the link to the electronic supplementary material.

Supplementary file1 (DOCX 21.2 KB)

## Data Availability

The data that support the findings of this study are available from the corresponding authors (DMS and AC).
