# Supplementary material for: Plasma SHBG Levels as an Early Predictor of Response to Bariatric Surgery
Source: Obes Surg. 2024 Jan 6;34(3):760–8. doi: 10.1007/s11695-023-06981-w (PMC10899416; doi:10.1007/s11695-023-06981-w)
Supplement: Supplementary file 1 — Supplementary file1 (DOCX 21.2 KB) [file 11695_2023_6981_MOESM1_ESM.docx]

**Supplementary table 1** Surgical procedure performed according to T2D status pre-BS:

| **Surgical procedure** | **T2D patients pre-BS** | **Non-T2D patients pre-BS** | **P value** |
| --- | --- | --- | --- |
| **Sleeve gastrectomy** | 12 | 16 | 0.712 |
| **RYGB** | 13 | 21 |  |

T2D: Type 2 Diabetes; BS: bariatric surgery; RYGB: Roux-en-Y-gastric bypass.

**Supplementary table 2** Mean of SHBG increase on the 1^st^ month after BS according to surgical procedure performed:

| **Surgical procedure** | **SHBG 1^st^ month / SHBG pre-BS ratio** | **Percentage** | **P value** |
| --- | --- | --- | --- |
| **Sleeve gastrectomy** | 1.59 (0.89) | 59% | 0.118 |
| **RYGB** | 1.94 (0.75) | 94% |  |

Values are mean (SD) and percentages.

SHBG: Sex hormone-binding globulin; RYGB: Roux-en-Y-gastric bypass.
